# Supplementary material for: How Do Patients with Life-Limiting Illness and Caregivers Want End-Of-Life Prognostic Information Delivered? A Pilot Study
Source: Healthcare (Basel). 2021 Jun 22;9(7):784. doi: 10.3390/healthcare9070784 (PMC8303293; doi:10.3390/healthcare9070784)
Supplement: Supplementary file 1 [file healthcare-09-00784-s001.zip › Supplement 1. survey questions with instructions.pdf]

Supplement 1. Survey questions with instructions

"Thank you for consenting to participate in this survey to assist us in deciding the best ways to communicate health information to patients in the future. This is an opinion survey so there are no right or wrong answers. We just want to find out general views on how patients and their families would like to receive information about their health conditions."

***Reminder to ask the participant if they will be answering the survey as their role as a current or past caregiver for someone who had/is suffering from a life-limiting condition or for themselves (can only answer as 'self' if suffering from a life-limiting condition)*

****If the participant does not have/had a life-limiting illness OR has not been a caregiver past or present to a relative/loved one than thank participant for their time and terminate interview.*

Data collection date	/ /
Respondents Role	1.☐ Caregiver<br>2. ☐ Self (if suffers from a life-limiting condition)
Participant Number	
Patient age group & sex *(if responding as caregiver question relates to their relative/loved one*)	Age group 1.☐ <70 years 2.☐ 70-79 years 3. ☐ 80+ years<br>Sex: 1.☐Male 2.☐Female
Country of birth (of participant)<br>____________________	What is your Highest level of Education? (of participant)<br>1.☐Primary 2.☐Secondary<br>3.☐Trade/technical 4.☐Tertiary
Participant Age and Sex	Age group 1.☐ <70 years 2.☐ 70-79 years 3. ☐ 80+ years<br>Sex: 1.☐Male 2.☐Female
1. Setting	1. ☐ Consumer phone interview 2. ☐Other (e.g., face-to-face)
2. SELF: In the last 12 months have you been admitted to hospital or presented to an emergency department?<br>OR<br>2. CAREGIVER: In the last 12 months (or if deceased in the last year of life) did your loved one present to an emergency department or admission to hospital?	1.☐ Neither<br>2. ☐ Yes presented to an Emergency Department w/out admission<br>3. ☐ Yes had at least one hospital admission<br>4. ☐ Both ED and at least one hospital admission<br>5. ☐ Does not remember

Preferred format and depth of prognostic information

3. SELF: Thinking generally about your health if you have a life-limiting illness **how much would you like to find out about your prognosis if you were to be consulted about it**<br><br>3. CAREGIVER: Thinking generally about your relatives/loved ones health if they have a life-limiting illness **how much would you like to find out about their prognosis if you were to be consulted about it**	1.☐Full information<br>2.☐A fair bit<br>3.☐Only a little<br>4.☐Not at all – I'll leave it to the experts<br>5.☐Not at all – I'm an expert at my own disease<br><br>**Why?**<br>____<br>____<br>____
**4. What aspect of prognostic information from the treating clinician(s) would you be interested in learning about?**<br><br>*(tick as many as relevant)*<br><br>***Do not prompt***	1.<br>☐Other____<br>____<br>____<br>2.☐Benefits of treatment<br>3.☐Possible treatment harms or side effects<br>4.☐Chances of cure with treatment<br>5.☐Impact of treatment on your quality of life<br>6.☐Probability of complications in the next 6 months<br>7.☐Impact of management on family or caregivers<br>8.☐Cost of treatment<br>9.☐ Results with other treatment alternatives<br>10.☐Expected survival time
5. If you like to know about benefits or harms of treatment or chances of cure, or probabilities of complications, **How would you like the information presented to you?**<br><br>*(tick as many as relevant but ask for their main preference)*	1. ☐ Verbal information from the doctor<br>2. ☐A picture of patient undergoing a treatments<br>3. ☐A table with numbers on probabilities based on previous studies<br>4. ☐Some graph that illustrates good and bad consequences<br>5. ☐Video supported explanation of the treatment<br><br>6. ☐Other<br><br>____<br><br>Combination of preference(s) No _____ (if participant cannot decide on one only)
6. What **format** would you prefer as the delivery method of this information? *(tick as many as relevant)*	1. ☐Paper<br>2. ☐Digital<br>3. ☐Paper & Digital<br>3. ☐Just verbal

<table>
<tr><td colspan="6">(For phone interviews – “You were emailed four scenarios which you should have with you and a YouTube link. Please do not view this until prompted”)</td></tr>
<tr><td>Scenario Order Sequence (refer to excel randomisation document)</td><td>5</td><td>2</td><td>3</td><td>4</td><td>1</td></tr>
<tr><td colspan="6">Note: 1=Verbal Information 2=Graphs 3=Table with numbers and % 4= Picture of treatment in ICU 5= CPR video
(Please note that the consumers DO NOT have the scenarios numbered. Read out each scenario e.g., "please look at the scenario with the picture of what treatment a patient in ICU is receiving)</td></tr>
<tr><td>7. Preference to scenario verbal only</td><td colspan="5">Do not like it at all Likes it strongly
__________1_______________2_________3_________4_________5____</td></tr>
<tr><td>8. Comment on reason for preference</td><td colspan="5">____
____</td></tr>
<tr><td>9. How distressing did you find this scenario</td><td colspan="5">Not at all Very distressing
_______1_______________2_________3_________4___ __________5____</td></tr>
<tr><td>10. Preference to scenario Numbers and % in a table</td><td colspan="5">Do not like it at all Likes it strongly
__________1_______________2_________3_________4_________5____</td></tr>
<tr><td>11. Comment on reason for preference</td><td colspan="5">____
____</td></tr>
<tr><td>12. How distressing did you find this scenario</td><td colspan="5">Not at all Very distressing
_______1_______________2_________3_________4___ __________5____</td></tr>
<tr><td>13. Preference to scenario Photo of patient in ICU</td><td colspan="5">Do not like it at all Likes it strongly
__________1_______________2_________3_________4_________5____</td></tr>
<tr><td>14. Comment on reason for preference</td><td colspan="5">____
____</td></tr>
<tr><td>15. How distressing did you find this scenario</td><td colspan="5">Not at all Very distressing
_______1_______________2_________3_________4___ __________5____</td></tr>
<tr><td>16.Preference to scenario information video on CPR (video link to be watched)</td><td colspan="5">Do not like it at all Likes it strongly
__________1_______________2_________3_________4_________5____</td></tr>
<tr><td>17. Comment on reason for preference</td><td colspan="5">____
____</td></tr>
<tr><td>18. How distressing did you find this scenario</td><td colspan="5">Not at all Very distressing
_______1_______________2_________3_________4___ __________5____</td></tr>
</table>

19. Preference to **scenario Graphs**	Do not like it at all Likes it strongly<br>_____1_____2_____3_____4_____5___
20. Comment on reason for preference	____________________<br>____________________
21. How distressing did you find this scenario	Not at all Very distressing<br>_____1_____2_____3_____4___ _____5___
22. Do you have any general suggestions for other formats to receive health information pertinent to terminal illness?	____________________<br>____________________<br>____________________<br>____________________

<u>Additional Comments/Free writing space</u>

(If the participant was wanting to add anything further)

"Thanks again for your time participating in this consultation"
