# Supplementary material for: How Do Patients with Life-Limiting Illness and Caregivers Want End-Of-Life Prognostic Information Delivered? A Pilot Study
Source: Healthcare (Basel). 2021 Jun 22;9(7):784. doi: 10.3390/healthcare9070784 (PMC8303293; doi:10.3390/healthcare9070784)
Supplement: Supplementary file 1 [file healthcare-09-00784-s001.zip › Supplement 2. Scenarios.pdf]

Supplement 2. Five hypothetical clinical scenarios

Scenario – Verbal information with basic take-home messages

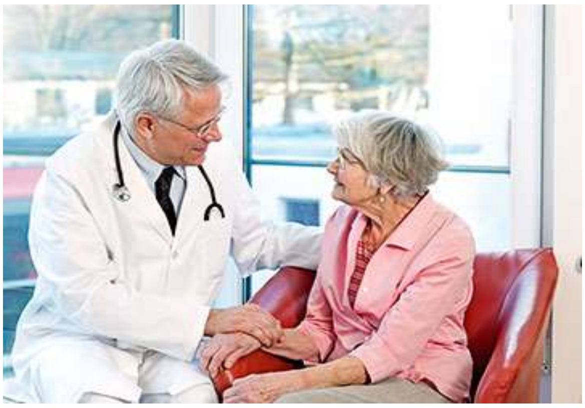

Visit Summary

Key points we discussed today:

-Your diagnosis is chronic obstructive pulmonary disease

-Your oxygen saturation is below 90% most of the day

-Your lung capacity is severely reduced to 50% of normal volume in an adult

Treatment options for you and outlook:

-A therapist can teach you techniques to reduce your breathing effort.

-This won't extend your life, but it will help enhance your quality of life.

- In end-stage COPD you'll likely need supplemental oxygen to breathe and you may not be able to complete activities of daily living.

- If your lung capacity is less than 35% you may not survive beyond four years after this result.

Instructions:

- Use the nicotine patches to help you stay off smoking

-Try to maintain your weight; do not lose weight because you will become more frail and less able to mobilise yourself.

-If your shortness of breath is hard to manage or you cannot walk too far in six minutes this means your COPD has progressed, and you may benefit from additional palliative or hospice care.

Contact Nurse XXX or Doctor YYY if you have any further questions

Treating doctor Date

Scenario – Table with data on prognosis with or without treatment at various times

Table 3

Mortality Rates at Various Time Points[a]

	% (Absolute No.)			
**Mortality Rate**	**Total (n=70)**	**Nonoperative (n=46)**	**Operative (n=24)**	***P***
In-hospital	5.7 (1.9)	6.5 (3)	4.2 (1)	1.000
3 mo	7.1 (5)	8.7 (4)	4.2 (1)	.654
6 mo	11.4 (8)	13.0 (6)	8.3 (2)	.706
1 y	12.9 (9)	15.2 (7)	8.3 (2)	.709
1 y postdischarge[b]	7.6 (5)	9.3 (4)	4.3 (1)	.651

[a]Represents the mortality rate of patients discharged after the initial injury.

Scenario – Pictures of what treatment will look like; e.g. patient in intensive care

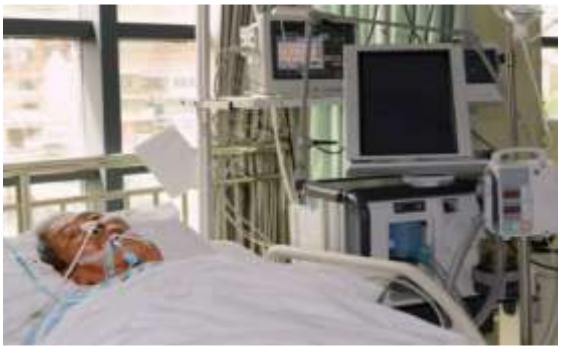

Scenario– Graphs of responses to chemotherapy and survival with different treatment

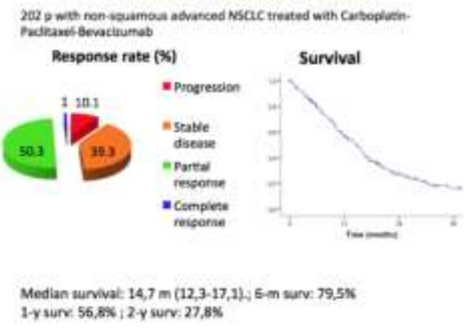

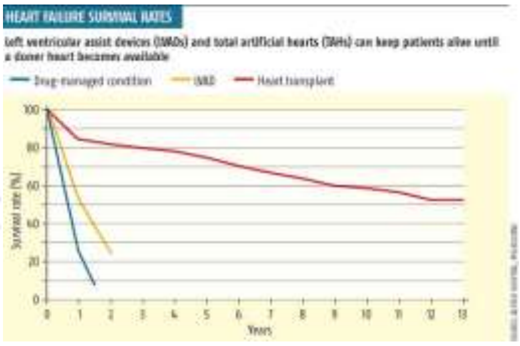

Scenario – Video of procedures and their outcomes

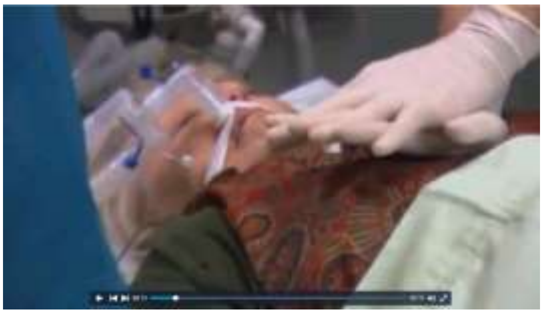

https://m.youtube.com/watch?v=j7bYRIQbw_g&list=PLvp5I1SjNZbMbgO7iys-Ta2j3GliLvYXl
